# Progress of The Journal of Functional Morphology and Kinesiology in 2019

**DOI:** 10.3390/jfmk5010003

**Published:** 2020-01-16

**Authors:** Giuseppe Musumeci

**Affiliations:** Department of Biomedical and Biotechnological Sciences, Anatomy, Histology and Movement Sciences Section, School of Medicine, University of Catania, Via S. Sofia 87, 95123 Catania, Italy; g.musumeci@unict.it

## 1. Looking Back on 2019

The *Journal of Functional Morphology and Kinesiology* (*JFMK*, ISSN: 2411-5142), which was firstly released in March 2016 [1], developed greatly in 2019. This journal provides an advanced forum for research studies on functional morphology and kinesiology and the regulatory functions of movement. *JFMK* meets the growing demand for high-quality, peer-reviewed international journals, supplying easy access, high publicity of Open Access, a digital object identifier (DOI), an ORCID, and CrossRef to all researchers. *JFMK* is indexed in the databases Scopus (Elsevier’s abstract and citation database), Directory of Open Access Journals (DOAJ), Scilit (a comprehensive, open-access scholarly database, developed and maintained by MDPI), as well as in Google Scholar’s, World Health Organization’s Hinari, and Food Science and Technology Abstracts (FSTA)’s (produced by IFIS) databases and in the Norwegian Register for Scientific Journals, Series, and Publishers (NSD). Our full texts are archived in CLOCKSS (digital archive), e-Helvetica (Swiss National Library Digital Archive), and J-Gate (Informatics India). We hope we can be included in Web of Science and PubMed in the near future, considering that six *JFMK* papers are already indexed in PubMed (https://www.ncbi.nlm.nih.gov/pubmed/?term=jfmk). *JFMK* is a member of the Committee on Publication Ethics (COPE). To verify the originality of content submitted to our journal, we use iThenticate and compare submissions with previous publications. MDPI works with Publons to provide reviewers with credit for their work and MDPI Scitations Alert to provide our authors information on new publications in their research field. 

*JFMK* publishes articles focusing on molecular, cellular, tissue, system, and whole-body responses to broadly defined physical activity. Furthermore, it provides an advanced forum for the analysis of the structure, function, development, and evolution of cells and tissues of the musculoskeletal system and associated disorders.

We are proud to let you know that, thanks to your continuous support, *JFMK* has continued to grow in the fields of morphology, kinesiology, movement, biomechanics, sport medicine, and musculoskeletal disorders. It is my pleasure to confirm the progress recorded in the last three years [2,3,4] as stated in our statistics https://www.mdpi.com/journal/jfmk/stats. 

Indeed, the number of published manuscripts has jumped from 55 in the 2018 volume to 63 in the 2019 volume, and we rejected in total 51 contributions to maintain the high standards of our journal. *JFMK* receives more manuscripts than it is able to publish, and the decision as to which papers are accepted or rejected is a difficult one. This decision is based on several factors, including originality, experimental design, scientific quality, data interpretation, clarity, and English quality, to maintain the high standards of our journal.

In 2019, various Special Issues were published thanks to the huge support of our editors. They include the following: “Psychology of Development and Education Applied to Movement”, edited by Prof. Dr. Marianna Alesi and Dr. Sebastiano Costa [5]; “Selected Papers from Mind in Motion—Physical Activity and Neuroscience”, edited by Prof. Dr. Marinella Coco [6]; “Health Promotion in Children and Adolescents through Sport and Physical Activities—2nd Edition”, edited by Prof. Antonino Bianco [7]; “Role of Exercises in Musculoskeletal Disorders—2nd Edition”, edited by Prof. Dr. Giuseppe Musumeci [8]; “TMJ Dysfunctions and Systemic Correlations”, edited by Dr. Luca Fiorillo [9]; “Research on Sports Nutrition: Body Composition and Performance”, edited by Prof. Dr. Jose Antonio [10]; “Resistance Training for Performance and Health—2nd Edition”, edited by Prof. Dr. Antonio Paoli [11]; “New Advances in Human Posture and Movement 2.0”, edited by Prof. Dr. Olivier Hue [12]; “Tailored Exercise in Patients with Chronic Diseases 2020”, edited by Prof. Dr. Laura Stefani [13]; “Overtraining Prevention”, edited by Prof. Dr. Maria Francesca Piacentini [14]; “Applied Sport Physiology and Performance”, edited by Dr. William Guyton Hornsby III [15]; “Exercise and Neurodegenerative Disease”, edited by Dr. Grazia Maugeri and Prof. Dr. Velia D’Agata [16]; “Performance Analysis and Training Monitoring in Team Sports”, edited by Prof. Dr. Antonio Tessitore [17]; “Fatigue and Motor Performance: A Way to Understand How the Human Body Adapts to Exercise”, edited by Dr. Helmi Chaabene and Prof. Dr. Olaf Prieske [18].

In 2019, three distinguished scientists joined the Editorial Board: Prof. Dr. Angela Lucariello (University of Naples “Parthenope”, Naples, Italy), Dr. Grazia Maugeri (University of Catania, Catania, Italy), and Prof. Michele Vecchio (University of Catania, Catania, Italy), for a total of 77 editorial board members and 7 advisory board members, besides the editor-in-chief.

Since 2019, all articles appearing in *JFMK* are published in open access; in order to provide free access to readers and to cover the costs of peer review, copyediting, typesetting, long-term archiving, and journal management, an article processing charge (APC) of 1000 CHF (Swiss Francs) is applied to papers accepted after peer review. 

## 2. Looking Forward to 2020

In 2020, we shall continue our efforts to improve the journal through further growth and increased visibility. 

In order to achieve this target and lay a strong foundation for high-quality publications and application for indexing in 2020, we have made the plans reported below. We will:

- Make sure that planned papers are followed up by editorial board members;
- Contact international conferences recommended by the editor-in-chief or by editorial board members and try to establish media partnerships with them to make *JFMK* increasingly well-known among scholars;
- Communicate with editorial board members regularly and ask for their help and suggestions for the journal’s development;
- Post high-quality papers through social media (e.g., LinkedIn, Twitter, and Facebook) and increase online readership;
- Reduce the processing time of each manuscript;
- Try to have publications indexed by the Emerging Sources Citation Index (Web of Science), by EMBASE (Elsevier) and by PubMed;
- Try to be included in the SCImago Journal Rank in the kinesiology-related section;
- Launch, for our authors, the “best *JFMK* paper” award and the “*JFMK* travel grant” award;
- Garner, for the sake of journal promotion, support from sponsors for our editors to participate in and disseminate our journal to international conferences.

We hope that you share our enthusiasm for this new journal and look forward to working with you to make *JFMK* a leader in its field. Your contributions are vital for the success of this new journal. We look forward to receiving your contributions (papers, reviews, etc.) as well as proposals for Special Issues.

It is my pleasure to end this editorial by wishing you a healthy and prosperous new year. This is also the opportunity for me to warmly thank, for their confidence, the following: our authors, readers, and reviewers, as well as our editorial advisors, eminent scientists in this field, who with their experience and important suggestions, are guiding us in this great enterprise; our excellent editorial board members, whose depth of experience covers a very broad spectrum in different disciplines related to morphology and kinesiology; the managing editor Olivia Yu for her huge support, the publishing manager Peter Roth, the assistant managing editor Molly Lu, and the assistant editor Sydney Tang, who day after day, with their valuable contributions, ensure the growth of this journal; finally, all members of our teams in Basel, Barcelona, Beijing, Belgrade, Romania, Tokyo, and Wuhan, as well as our sponsors.
